# Inhibiting the CB1 receptor in CIH-induced animal model alleviates colon injury

**DOI:** 10.1007/s00253-024-13216-0

**Published:** 2024-06-18

**Authors:** Pei-Pei Wang, Xiao-Qian Cheng, Zhan-Jun Dou, Yong-Qiang Fan, Jie Chen, Li Zhao, Jian-Xing Han, Xian-Wang Lin, Bei Wang

**Affiliations:** 1 Department of Respiratory, The Second Hospital of Shanxi Medical University, Taiyuan, China; 2 Department of Respiratory, Shanxi Cancer Hospital, Taiyuan, China; 3 Department of General Surgery, The First Hospital of Shanxi Medical University, Taiyuan, China; 4 Department of Nephrology, The Second Hospital of Shanxi Medical University, Taiyuan, China; 5 Department of Stomatology, The Second Hospital of Shanxi Medical University, Taiyuan, China

**Keywords:** Obstructive sleep apnea, Chronic intermittent hypoxia, CB1 receptor, Colon injury, Intestinal flora, Metabolic endotoxemia

## Abstract

**Abstract:**

Obstructive sleep apnea (OSA) can lead to intestinal injury, endotoxemia, and disturbance of intestinal flora. Additionally, as a crucial component of the endocannabinoid system, some studies have demonstrated that cannabinoid 1 (CB1) receptors are closely linked to the multiple organ dysfunction triggered by OSA. However, the role of the CB1 receptor in alleviating OSA-induced colon injury remains unclear. Here, through the construction of the OSA classic model, we found that the colon tissue of chronic intermittent hypoxia (CIH)–induced mice exhibited an overexpression of the CB1 receptor. The results of hematoxylin-eosin staining and transmission electron microscopy revealed that inhibition of the CB1 receptor could decrease the gap between the mucosa and muscularis mucosae, alleviate mitochondrial swelling, reduce microvilli shedding, and promote the recovery of tight junctions of CIH-induced mice. Furthermore, CB1 receptor inhibition reduced the levels of metabolic endotoxemia and inflammatory responses, exhibiting significant protective effects on the colon injury caused by CIH. At the molecular level, through western blotting and real-time polymerase chain reaction techniques, we found that inhibiting the CB1 receptor can significantly increase the expression of ZO-1 and Occludin proteins, which are closely related to the maintenance of intestinal mucosal barrier function. Through 16S rRNA high-throughput sequencing and short-chain fatty acid (SCFA) determination, we found that inhibition of the CB1 receptor increased the diversity of the microbial flora and controlled the makeup of intestinal flora. Moreover, butyric acid concentration and the amount of SCFA-producing bacteria, such as *Ruminococcaceae* and *Lachnospiraceae*, were both markedly elevated by CB1 receptor inhibition. The results of the spearman correlation study indicated that *Lachnospiraceae* showed a positive association with both ZO-1 and Occludin but was negatively correlated with the colon CB1 receptor, IL-1β, and TNF-α. According to this study, we found that inhibiting CB1 receptor can improve CIH-induced colon injury by regulating gut microbiota, reducing mucosal damage and promoting tight junction recovery.

**Key points:**

*•CIH leads to overexpression of CB1 receptor in colon tissue.*

*•CIH causes intestinal flora disorder, intestinal mucosal damage, and disruption of tight junctions.*

*•Inhibition of CB1 receptor can alleviate the colon injury caused by CIH through regulating the gut microbiota, reducing mucosal injury, and promoting tight junction recovery.*

**Graphical abstract:**

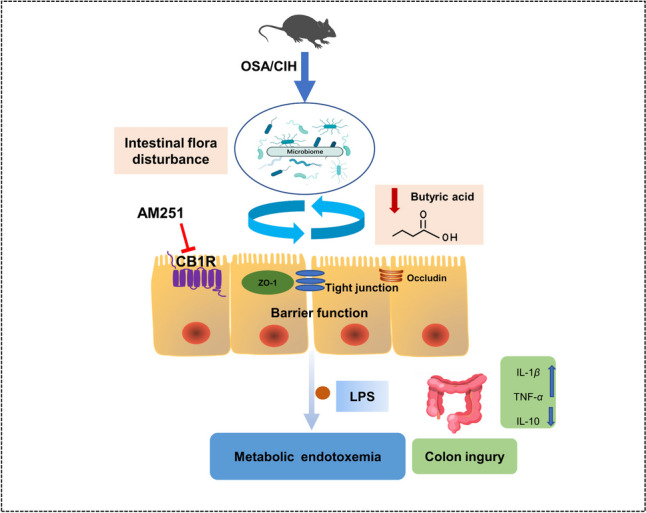

## Introduction

Obstructive sleep apnea (OSA) is a common sleep condition marked by repeated collapse of the upper respiratory tract that interferes with normal breathing patterns during sleep. OSA can lead to chronic intermittent hypoxia (CIH), sympathetic excitation, and sleep fragmentation due to upper respiratory tract collapse. Among these characteristics, the most serious consequence of OSA is the induction of CIH, resulting in a sustained state of reduced oxygen levels across the body (Myers et al. 2013). Increasing researches have pointed out that OSA is closely related to the occurrence and development of diabetes, hypertension, cognitive dysfunction, cancers, and other diseases (Drager et al. 2013; Kheirandish-Gozal and Gozal 2017; Yeghiazarians et al. 2021; Sánchez-de-la-Torre et al. 2023). However, the key mechanism has not been clarified and further exploration is still needed.

The intestinal barrier constitutes the interaction between the human environment and the external world. The semipermeable membrane property of intestinal structure provides a cornerstone for exchange of substances inside and outside the intestine, absorption of nutrients, maintenance of body homeostasis, and immune surveillance and response (Suzuki 2020; Allam-Ndoul et al. 2020). The composition of the intestinal barrier is very complex, including intestinal flora, mucous layer, epithelial cell layer, immune cells in the lamina propria, and tight junction proteins ZO-1 and Occludin responsible for regulating the paracellular space of epithelial cells (Allam-Ndoul et al. 2020; Di Tommaso et al. 2021). According to relevant research, it has been observed that CIH leads to a decrease in the intestinal mucosal goblet cells, reduction in the tight junction proteins, increase in the markers associated with impaired intestinal barrier, as well as alterations in the abundance and diversity of gut microbiota (Wang et al. 2022b; Li et al. 2023). The integrity of intestinal barrier is subsequently compromised.

The endocannabinoid system is a signaling system composed of endocannabinoids, cannabinoid receptors, and various proteins involved in metabolism. The primary endocannabinoids are N-arachidonic ethanolamine and 2-arachidonic glycerol, and CB1 and CB2 receptors are the two most widely studied cannabinoid receptors at present (Iannotti et al. 2016). CB1 receptors are distributed in the central nervous system, liver, skeletal muscle, kidney, and digestive tract, while CB2 receptors are primarily distributed in the peripheral immune system. Previous studies have shown that the expression of CB1 receptor increased in multiple tissues, such as kidney, bone, and brain, under CIH conditions. Meanwhile, CB1 receptor antagonist could alleviate organ damage caused by CIH (Gao et al. 2018; Dou et al. 2020; Zhao et al. 2021). It therefore suggests that the endocannabinoid system is involved to the organ damage caused by OSA.

Multiple researches have found that the maintenance of intestinal barrier is regulated through the cannabinoid system. Recent studies have shown that activating cannabinoid receptors through phytocannabinoid can restore the integrity of the intestinal barrier by promoting the expression of tight junction proteins, facilitating mucus secretion, improving intestinal flora disorders, and inhibiting inflammation (Alhamoruni et al. 2012; Becker et al. 2021). However, the impact of the cannabinoid system on the intestinal barrier and its mechanism are still controversial. Because some studies have pointed out that the activation of CB1 receptor can lead to the destruction of the intestinal barrier (Mehrpouya-Bahrami et al. 2017). Meanwhile, another study also demonstrated that the CB1 receptor antagonist can further prevent the pathway of lipopolysaccharide (LPS) from gut to circulation. Compared with the control group, the decrease of serum LPS in the CB1 receptor antagonist group indicates further repair of the intestinal barrier (Muccioli et al. 2010). As mentioned above, the multi-organ damage caused by CIH is related to the abnormal expression of CB1 receptor, and cannabinoid receptors are closely related to the integrity of the intestinal barrier. Therefore, whether CB1 receptor is the key mechanism of intestinal barrier damage caused by CIH still needs further clarification. No previous studies have explored the effects of antagonists on intestinal barrier impairment caused by CIH. We propose the hypothesis that CIH exposure may lead to the disruption of intestinal barrier integrity and the occurrence of intestinal flora disorder, and that the use of CB1 receptor antagonist can reverse these effects. We discovered for the first time that inhibition of CB1 receptor can alleviate the colon injury caused by CIH through regulating the gut microbiota, reducing mucosal injury, and promoting tight junction recovery.

## Materials and methods

### Animal grouping and intervention

The treatment of experimental animals is in accordance with Chinese ethical requirements (Ethics No. :SYDL20200011). Male C57BL/6 mice between the ages of 4 and 5 weeks were obtained from the Laboratory Animal Center of Shanxi Medical University and maintained under specific pathogen free (SPF) environment with a normal diet (20.3% kcal from protein, 15.8% kcal from fat, and 63.9% kcal from carbohydrates). The mice were arbitrarily assigned into three groups (*n* = 6 for each group) as follows: (1) Control group, (2) CIH group, and (3) CIH group treated with CB1 receptor antagonist (AM251 group). Specifically, mice were subjected to intraperitoneal injection of the CB1 receptor antagonist AM251(1 mg/kg) before entering the intermittent hypoxic chamber. AM251 is a selective antagonist of the CB1 receptor, which has been widely used in various experiments. The inhibitory effect of AM251 on the CB1 receptor is significantly reliable (Sugawara et al. 2013; Caltana et al. 2015; Higginbotham et al. 2021). CIH-exposed mice were put into the intermittent hypoxic chamber at 9:00 am every day until 5:00 pm. The mice were kept under either a normal air or CIH environment for 6 weeks before the following experiments.

### Chronic intermittent hypoxia mouse model

CIH-exposed mice were placed in the OxyCycler Model A84 hypoxic chamber (Biospherix, Parish, NY, USA), which was set up in a mode of alternating 9–21% oxygen concentration and filled with high-purity compressed nitrogen and high-purity compressed oxygen for 120s per cycle. In the experimental setup, nitrogen was injected to reduce the oxygen concentration from 21% to 9% within 50s, and the hypoxia condition was maintained for 10s. Following this, high-purity oxygen is injected for 50s, rapidly raising the oxygen concentration back to 21%, which was then sustained for an additional 10s. The hypoxia-reoxygenation cycle was repeated 30 times every hour, with the lowest oxygen concentration being ± 9%, as previously described (Dou et al. 2020; Zhao et al. 2021). This protocol was carried out continuously for 8 h each day, from 9:00 am to 5:00 pm.

### Antibodies

CB1 receptor antagonist AM251 was purchased from Topscience (Shanghai, China, No: T1915), and rabbit anti-mouse Occludin (GB111402) and ZO-1 (GB11149) antibodies were purchased from Servicebio (Wuhan, China); rabbit anti-mouse CB1R antibody was purchased from Thermo Fisher (Waltham, MA, USA). Goat anti-rabbit second antibody was purchased from Wuhan Sanying (Wuhan, China).

### Hematoxylin-eosin (HE) staining

Colon tissues of mice were collected and fixed using 4% paraformaldehyde solution. Paraffin embedding of tissue was performed following fixation, then tissues were sectioned (5 µm). The sections were then dewaxed with conventional xylene, dehydrated with a gradient concentration of alcohol, and rinsed with PBS. HE staining was performed for 5 min, followed by dehydration using conventional gradient alcohol. The sections then underwent transparent process using xylene 2 times for 5 min each. Finally, the sections were sealed with neutral gum. An optical microscope was used to view the pathological changes in the colon tissue.

### Transmission electron microscopy (TEM)

Fresh colon tissues were trimmed into the size of 1 mm^3^ following collection. The tissues were then immediately placed in a centrifuge tube filled with 2.5% glutaraldehyde electron microscope fixative solution and stored at 4 ℃ for preservation. Subsequently, for 2h, the tissues were fixed in 1% osmic acid at room temperature. Dehydration was performed at room temperature, followed by permeation and polymerization. Ultrathin sections were prepared and stained accordingly. The ultrastructural changes within the colon tissue were studied through a transmission electron microscope. Images were captured and subsequently analyzed.

### Immunofluorescence staining

Paraffinized sections of the colon were dewaxed through rehydration, antigen was retrieved using the microwave oven, sealed with 3% bovine serum albumin (BSA) for 30 min, followed by drops of primary antibody CB1R (1:500, Proteintech, Wuhan, China), incubated overnight at 4 ℃, followed by drops of 100 μl anti-rabbit IgG labeled fluorescent antibody, incubated for 50 min at room temperature, nucleated with 4′,6-diamidino-2-phenylindole (DAPI) working solution at 37 ℃ for 10 min, thereafter sealed. Under a laser confocal microscope, the positive signal was red fluorescence and the blue was nuclear staining signal. The average fluorescence intensity of positive expression was calculated.

### 16S rRNA gene amplification and sequencing

At the end of the study (week 6), fecal samples were collected. Each group contains six mice. DNA of fecal samples was extracted and PCR amplification was performed using primers bound to the 16S rRNA highly variable region V3-V4. The community DNA fragments were paired with paired-end sequencing using an Illumina platform (Illumina, San Diego, CA, USA). The raw sequencing data was processed and analyzed on the QIIME2 analysis platform (Sun et al. 2023).

### Fecal SCFA analysis

Mouse feces were collected and stored at −80 ℃. The short-chain fatty acid (SCFA) test includes acetic acid, butyric acid, isovaleric acid, valeric acid, caproic acid, propionic acid, and isobutyric acid. A gas phase mass spectrometer was used for the detection. Conditions for chromatography were as follows: Thermo Trace 1310 gas phase system (Thermo Fisher Scientific, Waltham, MA, USA), Agilent HP-INNOWAX capillary column (Agilent, Santa Clara, CA, USA), shitter injection, injection volume 1 μL, shitter ratio 10:1, inlet temperature 250 ℃, ion source temperature 300 ℃, transmission line temperature 250 ℃. The starting temperature of programmed heating is 90 ℃. Then it was heated at 10 ℃/min to 120 ℃, then at 5 ℃/min to 150 ℃, then lastly at 25 ℃/min to 250 ℃ for 2 min. The carrier gas was helium, and the carrier gas flow rate was 1.0 mL/min. Mass spectrum conditions were as follows: Thermo ISQ LT Mass spectrometer (Thermo Fisher Scientific, Waltham, MA, USA), electron bombardment ionization (EI) source, selected ion monitor (SIM) scanning mode electron energy 70 eV.

### Western blotting

The protein from colon tissue was isolated and the bicinchoninic acid assay (BCA) was run to determine the concentration of the protein. The protein samples were denatured by water bath at 100 ℃ for 10 min prior to sodium dodecyl sulfate-polyacrylamide gel electrophoresis (SDS-PAGE). The membrane was transferred to a polyvinylidene fluoride (PVDF) membrane by electric transfer, blocked with skim milk, followed by overnight incubation with the preceding primary antibodies at 4 °C: Rabbit anti-ZO-1 (1:2000, Servicebio, Wuhan, China), rabbit anti-Occludin (1:3000, Servicebio, Wuhan, China), and rabbit anti-CB1 receptor (1:3000, Thermo Fisher, Waltham, MA, USA). Thereafter, membranes were washed with 0.05% Tween-20 (TBST) three times (5 min each). The membranes were then incubated with secondary antibodies at room temperature (Proteintech, Wuhan, China) for 2 h, followed by TBST rinses three times (5 min each). Development was performed using an enhanced chemical luminizer (Millipore, Boston, MA, USA) and the signal was visualized in a comprehensive gel imaging system (Bio-Rad, Hercules, CA, USA). β-Actin was used as an internal reference protein.

### Real-time polymerase chain reaction (RT-PCR)

Total RNA was isolated from frozen colonic tissue by the TRIzol method and reverse transcribed into cDNA (Kidd et al. 2005). Using the gene for GAPDH as an endogenous control, 2^-ΔΔCt^ was used to calculate the relative expression level of the target gene after the reaction. All primers used are listed in Table 1.

**Table 1:** The list of primers sequences

Gene	Forward	Reverse
*GAPDH*	GGTTGTCTCCTGCGACTTCA	TGGTCCAGGGTTTCTTACTCC
*CB1R*	TCGACAGGTACATATCCATTCACA	GAGAGGCAACACAGCGATTACTATT
*ZO-1*	GGAAACCCGAAACTGATGCTATGG	AACTGGCTGGCTGTACTGTGAG
*Occludin*	TCTCAGCCGGCATACTCTTT	ATAGGCTCTGTCCCAAGCAA
*IL-1β*	AGCTTCCTTGTGCAAGTGTCTG	GACCACTCTCCAGTACCCACT
*TNF-α*	CAGGCGGTGCCTATGTCTC	CGATCACCCCGAAGTTCAGTAG
*IL-10*	CCTCTGGATACAGCTGCGAC	TAGACACCTTTGTCTTGGAGCTA

### Enzyme-linked immunosorbent assay (ELISA)

Mice whole blood samples were obtained and centrifuged for 2 h at 3000 rpm in a 4 °C low-temperature centrifuge for 15 min. Serum (supernatant) was collected to measure the concentration of LPS according to the kit instructions (Jingmei, Yancheng, China).

### Statistical analysis

Data analysis program utilized was SPSS 26.0 (SPSS, Chicago, IL, USA), and GraphPad Prism 8 (GraphPad, San Diego, CA, USA) was used for plotting. One-way ANOVA was used for samples with normal distribution, LSD test was employed for comparison when variance within groups was homogeneous, and when variance was uneven, Dunnett T3 test was utilized. Samples that did not satisfy the normal distribution were compared between groups using Kruskal-Wallis non-parametric tests.* P* < 0.05 was judged as statistically significant.

## Results

### CIH promotes overexpression of CB1 receptor in colon tissue

**Fig. 1:**
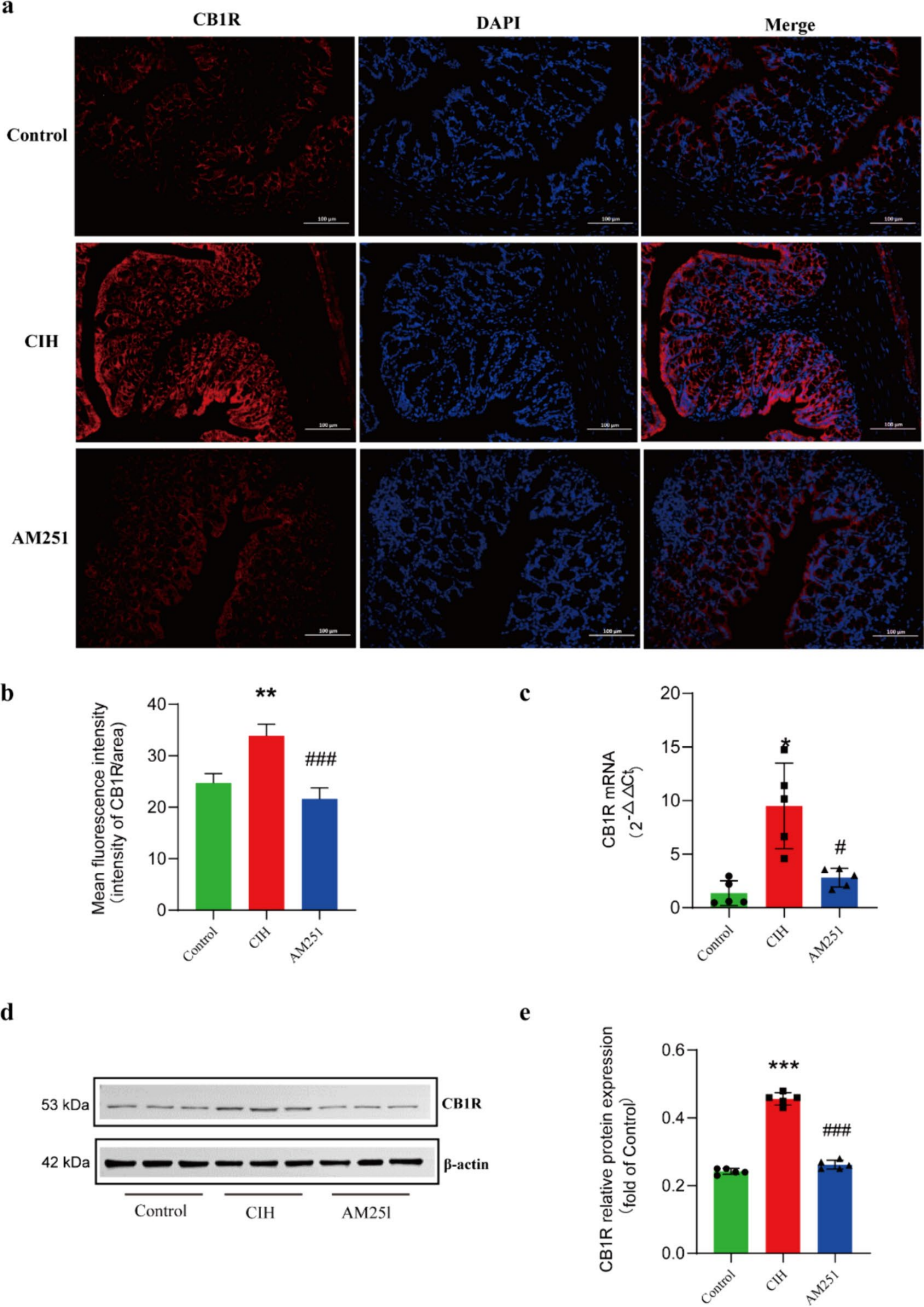
CIH promotes overexpression of CB1 receptor in colon tissue. **a-b** The expression of CB1 receptor in colon tissues was detected by immunofluorescence. **c** RT-PCR was used to detect the mRNA expression level of CB1 receptor in colon tissues. **d-e** The expression level of CB1 receptor protein in colon tissues was detected by western blot. *P < 0.05, **P < 0.01,***P < 0.001, compared with the Control group; ^#^*P* < 0.05, ^##^*P* < 0.01,^###^*P* < 0.001, compared with the CIH group

In Fig. 1, we examined the CB1 receptor expression levels in colon tissue. Immunofluorescence and western blot showed that CIH exposure promoted the high expression of the CB1 receptor, and AM251 treatment significantly decreased the level of the CB1 receptor. Similarly, RT-PCR revealed that the expression levels of the CB1 receptor in the CIH group were higher. After AM251 treatment, the mRNA level of the CB1 receptor was considerably reduced.

### Inhibition of CB1 receptor attenuates the pathological injury of colon tissue in CIH-exposed mice

**Fig. 2:**
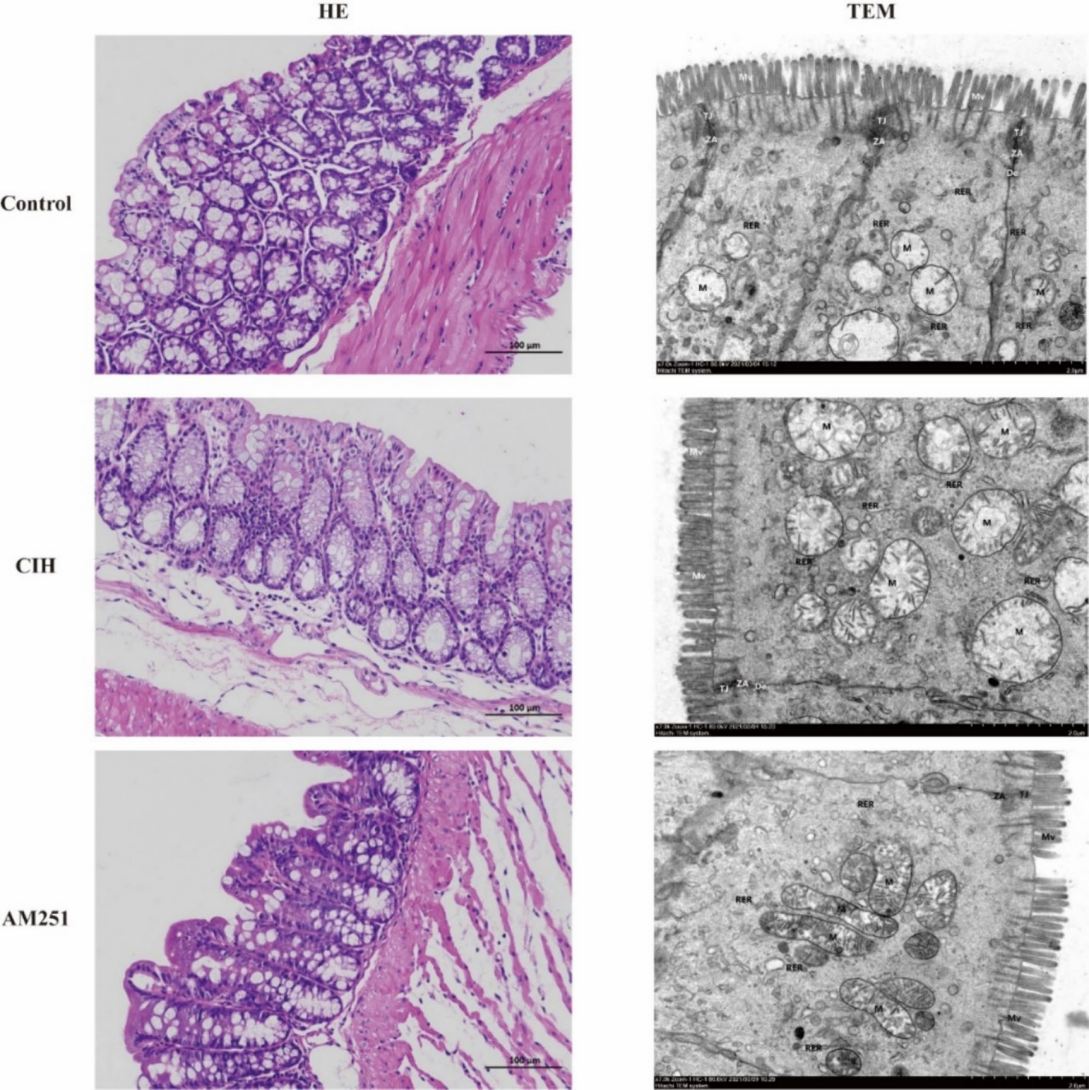
Inhibiting CB1 receptor mitigated the pathological damage of colon induced by CIH. Representative image of HE staining (100 µm) and transmission electron microscopy (20 µm) of colon tissue. TEM, transmission electron microscopy

As shown in Fig. 2, HE staining and TEM were utilized to examine the pathological morphological changes of colon tissue in mice. The HE staining results revealed that the distance between the mucosal midbowel gland and mucosal myometria increased and the CIH group was highly infiltrated by inflammatory cells in comparison to the Control group. In contrast, the degree of colon tissue damage and inflammatory cell infiltration was substantially reduced in the AM251 group in comparison to the CIH group. TEM revealed that as opposed to the Control group, mitochondria in the CIH group exhibited severe swelling, enlarged volume, dissolution of the membrane matrix, shortened and curled shape, reduced ridge structure, and partial vacuolation. In addition, there was a slight expansion of rough endoplasmic reticulum, shortened in tight junctions, blurred in dense regions, unclear in some intermediate junctions, and reduced in protein filaments in desmosome dense regions. However, we observed that mitochondrial swelling was reduced, microvilli were shed in a small range, and there were tight intercellular connections and intermediate connections upon inhibition of the CB1 receptor by AM251. According to these findings, the inhibition of the CB1 receptor may alleviate the pathological injury of colonic tissue in CIH-exposed mice.

### Inhibition of the CB1 receptor improves intestinal flora dysregulation in CIH-exposed mice

**Fig. 3:**
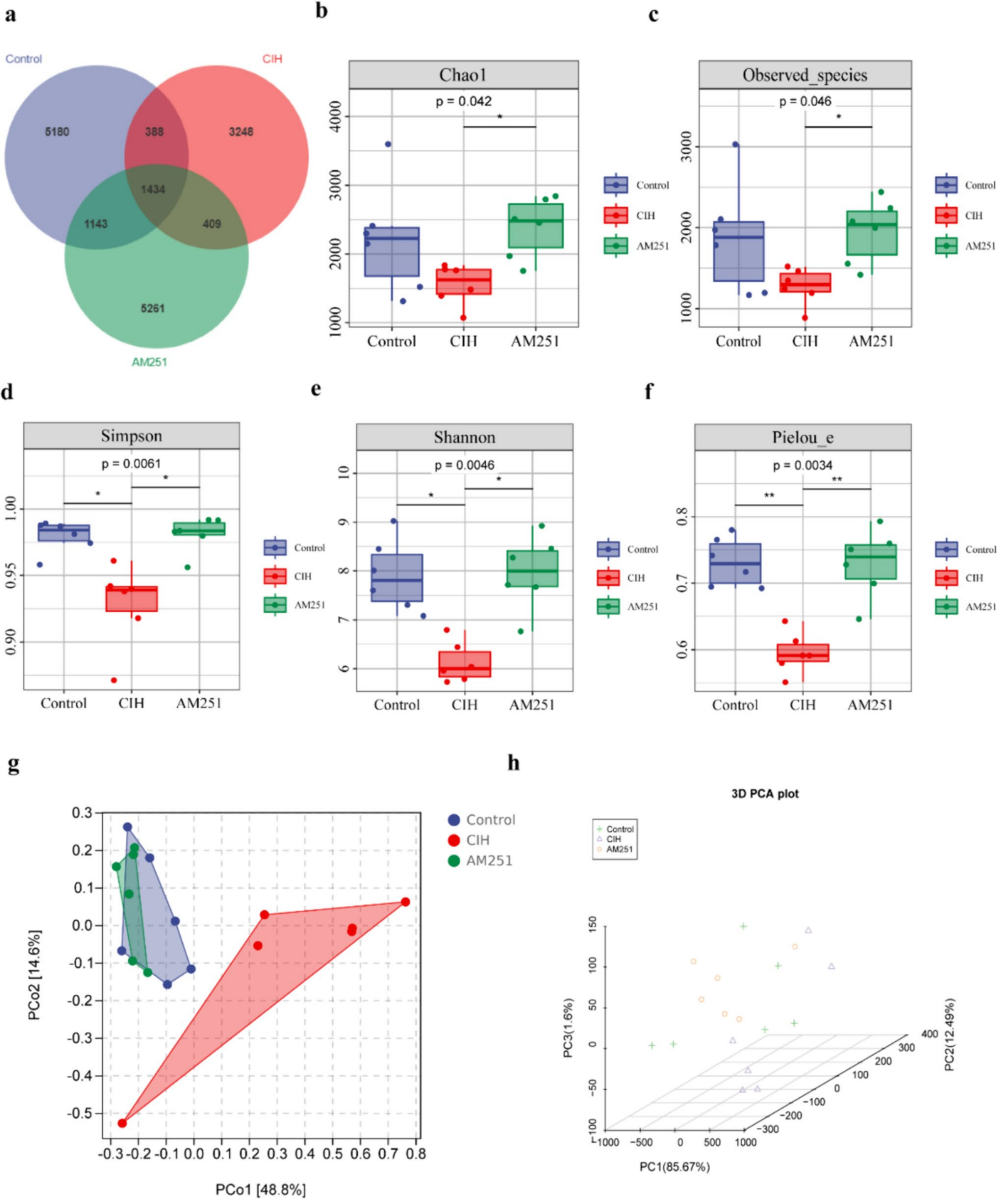
Effect of inhibiting CB1 receptor on intestinal microbiome of OSA mice. **a** OTU Wayne chart; **b**-**c** Chao1 and Observed_species indicate the richness of the community;** d**-**e** Shannon and Simpson indicate the diversity of the community; and **f** Pielou_e indicate the evenness of the community. **g** PCoA principal component analysis based on weighted_unfirc distance. **h** 3D. *P < 0.05, **P < 0.01

To investigate the consequence of inhibiting the CB1 receptor on the intestinal flora of CIH-exposed mice, we collected mouse feces and analyzed the makeup and function of the gut flora of mice using 16S rRNA gene V3-V4 region sequencing technology. A total of 1,010,596 valid sequences were obtained in this sample for the construction of operational taxonomic units (OTUs). OTU cluster analysis showed that 8145 OTUs were in the control group, 5479 OTUs were from the CIH group, 8247 OTUs were from the AM251 group, and 1434 overlapping OTUs were in all three groups (Fig. 3a). Species diversity analysis was used to study the changes of intestinal flora structure among all groups. The results of alpha diversity analysis showed that in the observed Chao1 and Observed_species index, the CIH group is comparable to the Control group; however, Simpson, Shannon, and Pielou_e indices were considerably decreased in the CIH group in comparison to the Control group (Fig. 3b–f). AM251 treatment can significantly increase alpha diversity index, including Chao1, Observed_species, Simpson, Shannon, and Pielou_e indices. These indicate that CIH exposure reduced specific bacterial groups, while AM251 treatment increased specific bacterial groups. Next, we studied the similarity of microbial communities through PCoA and 3D principal coordinate analysis. The weighted_unfirc distance for beta diversity analysis showed that the samples in the Control group were significantly clustered with those in the AM251 group, and completely separated from the samples in the CIH group; this suggests that the composition of the gut microbiota is markedly different in these three groups (Fig. 3g, h).

**Fig. 4:**
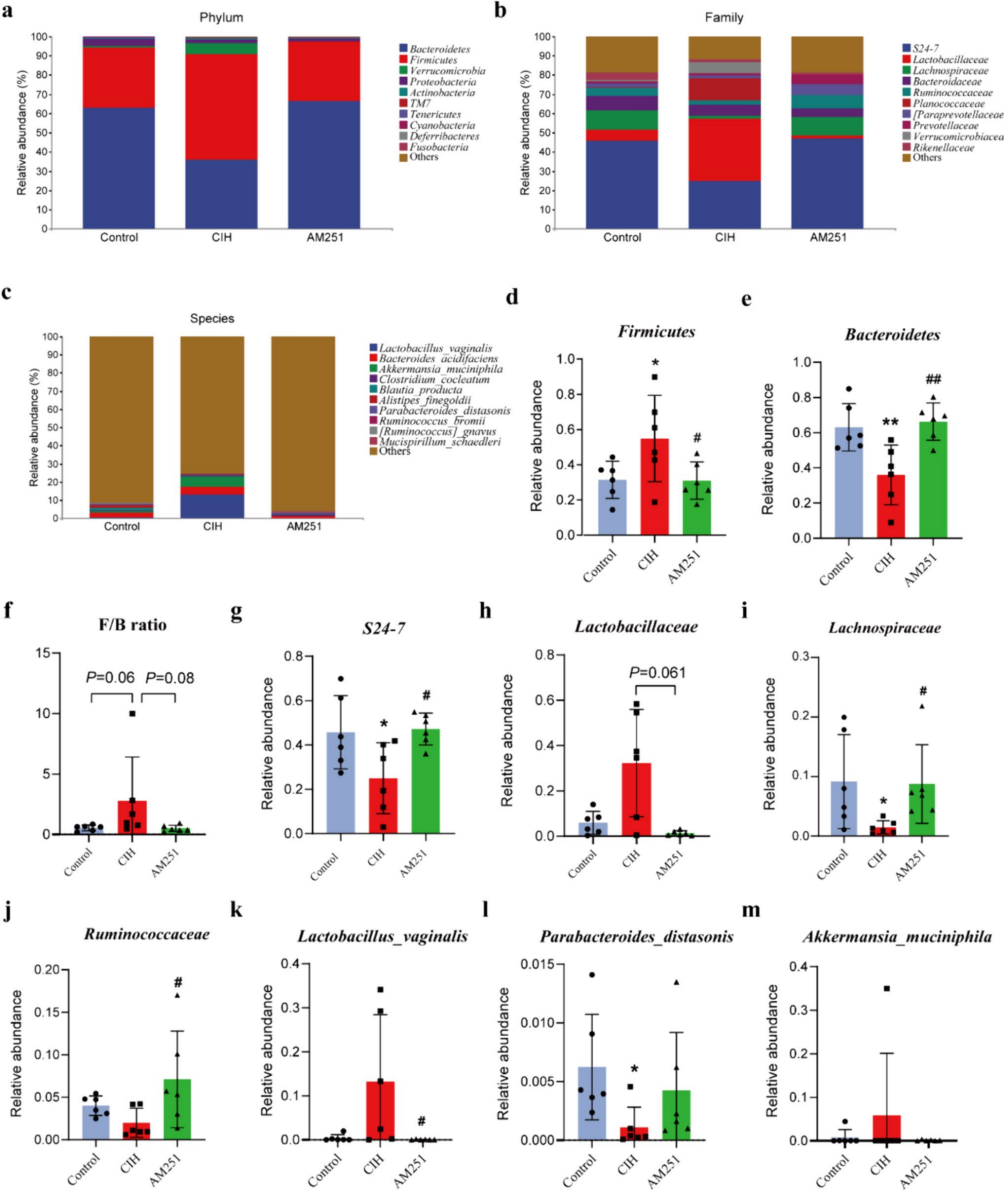
Inhibition of CB1 receptor alters the gut microbiome composition induced by CIH. **a** Intestinal microbial composition of top 10 phylum level. **b** The intestinal microbial composition of the top 10 families. **c** Intestinal microbial composition of top 10 species level. Difference analysis of flora at **d**–**f** phylum level, **g–j** family level, and **k**–**m** species level. *P < 0.05, **P < 0.01, compared to the Control group; ^#^*P* < 0.05, ^##^*P* < 0.01, compared to the CIH group

We further assessed the differences in microbiomes between the different groups. The relative abundances of the top 10 phylum, family, and species levels also changed significantly after CIH exposure (Fig. 4a–c). Notably, at the bacterial phylum level, as opposed to the Control group, the number of *Firmicutes* in the CIH group was significantly elevated, while the number of *Bacteroidetes* was significantly reduced, with a higher *Firmicutes/Bacteroidetes* (F/B) ratio. Inhibition of the CB1 receptor by its antagonist AM251 significantly decreased the quantity of *Firmicutes*, elevated the quantity of *Bacteroidetes*, and decreased the F/B ratio, indicating an improved composition of intestinal flora (Fig. 4d–f). At the family level, in comparison with the Control group, the numbers of *S24-7*, *Lachnospiraceae*, and *Ruminococcaceae* in CIH-exposed mice were reduced, whereas the quantity abundance of *Lactobacillaceae* was elevated. AM251 administration significantly elevated the quantity of *S24-7* and SCFA-producing bacteria *Lachnospiraceae* and *Ruminococcaceae* (Fig. 4g–j). At the species level, in comparison to the Control group, the number of *Lactobacillus_vaginalis* and *Akkermansia_muciniphila* increased in the CIH group, and the number of *Parabacteroides_distasonis* decreased significantly. Compared with the CIH group, AM251 treatment substantially decreased the quantity of *Lactobacillus_vaginalis* (Fig. 4k–m).

**Fig. 5:**
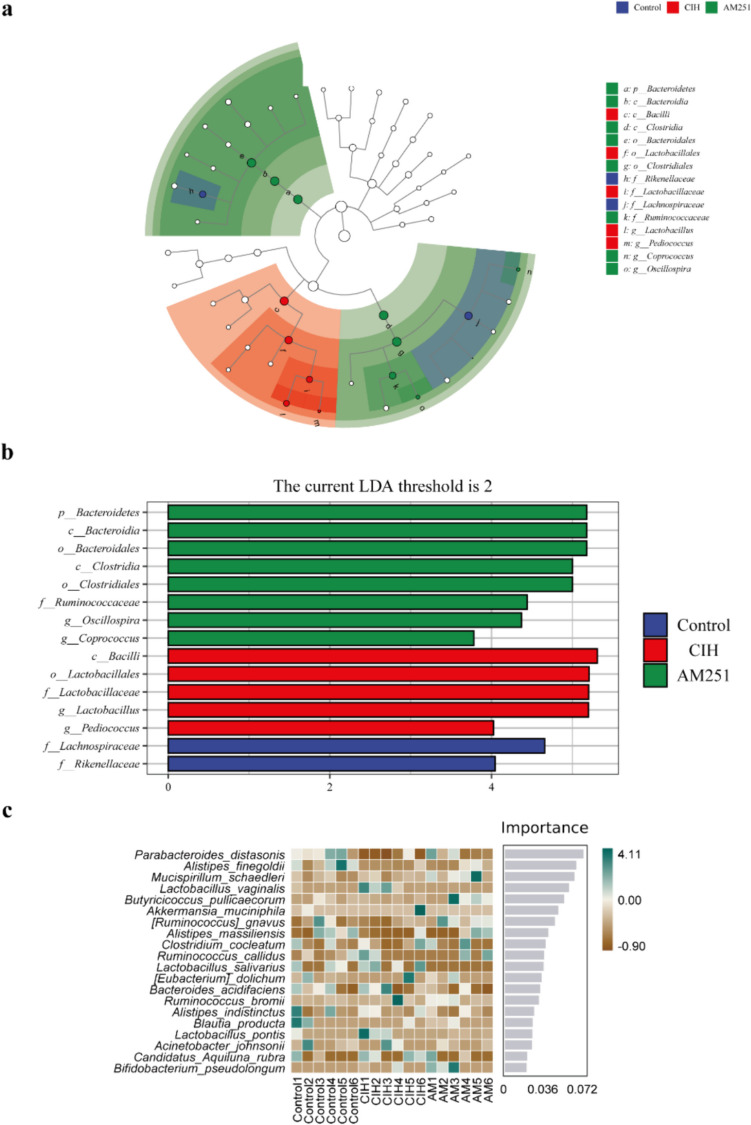
**a** LEFse branch diagram. **b** LEFse histogram analysis of marker species with significant differences between groups (LDA>2). **c** Random forest analysis: The heat map shows the abundance distribution of these species in each group. From top to bottom, the importance of species to the model decreases in order

Subsequently, LEfse analysis was used to identify marker species with significant differences between groups. The OTUs that significantly changed in the Control group included *f_Lachnospiraceae*, *f_Rikenellaceae*, and *s*__ __*Parabacteroides_distasonis*, while the OTUs that significantly changed in the CIH group included *c*__ __*Bacilli*, *o*____*Lactobacillales*, *f_Lactobacillaceae*, *g_Lactobacillus*, *s_Lactobacillus_vaginalis*, and *g*_--_*Prediococcus*, respectively. The OTUs that changed significantly in AM251 group were* p*__ __*Bacteroidetes*, *c_ Bacteroidia*, *o*__ __*Bacteroidales*, *c*__ __*Clostridia*, *o_Clostridiales*, *f*__ __*Ruminococcaceae*, *g_Oscillospira*, and *g_Coprococcus* (Fig. 5a, b). Meanwhile, we employed random forest analysis to describe species differences between groups. The species’ significance to the classifier model is displayed in the bar chart, and the heat map shows the abundance distribution of these species in each sample. It can be considered that the most important species among these species are markers of differences between groups. We could see a significantly higher prevalence of *Parabacteroides_distasonis* in the Control group and the AM251 group comparison to the CIH group (Fig. 5c).

### Role of CB1 receptor in metabolic pathways

**Fig. 6:**
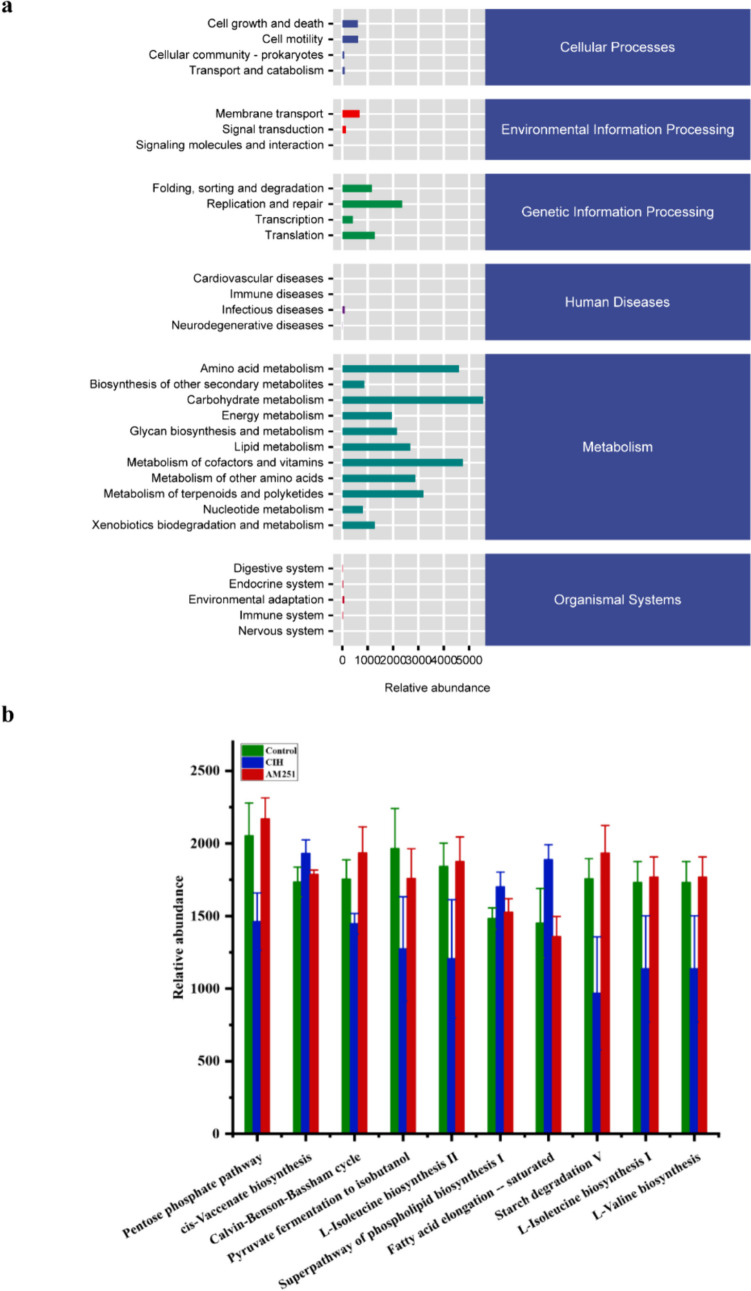
Correlation analysis of intestinal microbiota and metabolic pathways. **a** The relative abundance of metabolic pathways. **b** Top 10 components of KEGG metabolic pathways with significant differences among all groups

We utilized the Picrust2-generated KEGG functional pathway to predict potential functions associated with changes in the intestinal flora. The results show that intestinal flora are primarily enriched in cellular processes, environmental information processing, genetic information processing, human diseases, metabolism, and organismal systems functions (Fig. 6a). Next, to assess the influence of AM251 on the function of the gut flora, we further analyzed the top 10 significantly different metabolic pathways. Figure 6b demonstrates that the AM251 group was significantly enriched in the Pentose phosphate pathway, Calvin-Benson-Bassham cycle, Pyruvate fermentation to isobutanol, L-isoleucine biosynthesis II, Starch degradation V, L-isoleucine biosynthesis I, and L-valine biosynthesis in comparison to the CIH group.

### Effect of inhibiting CB1 receptor on fecal SCFA

**Fig. 7:**
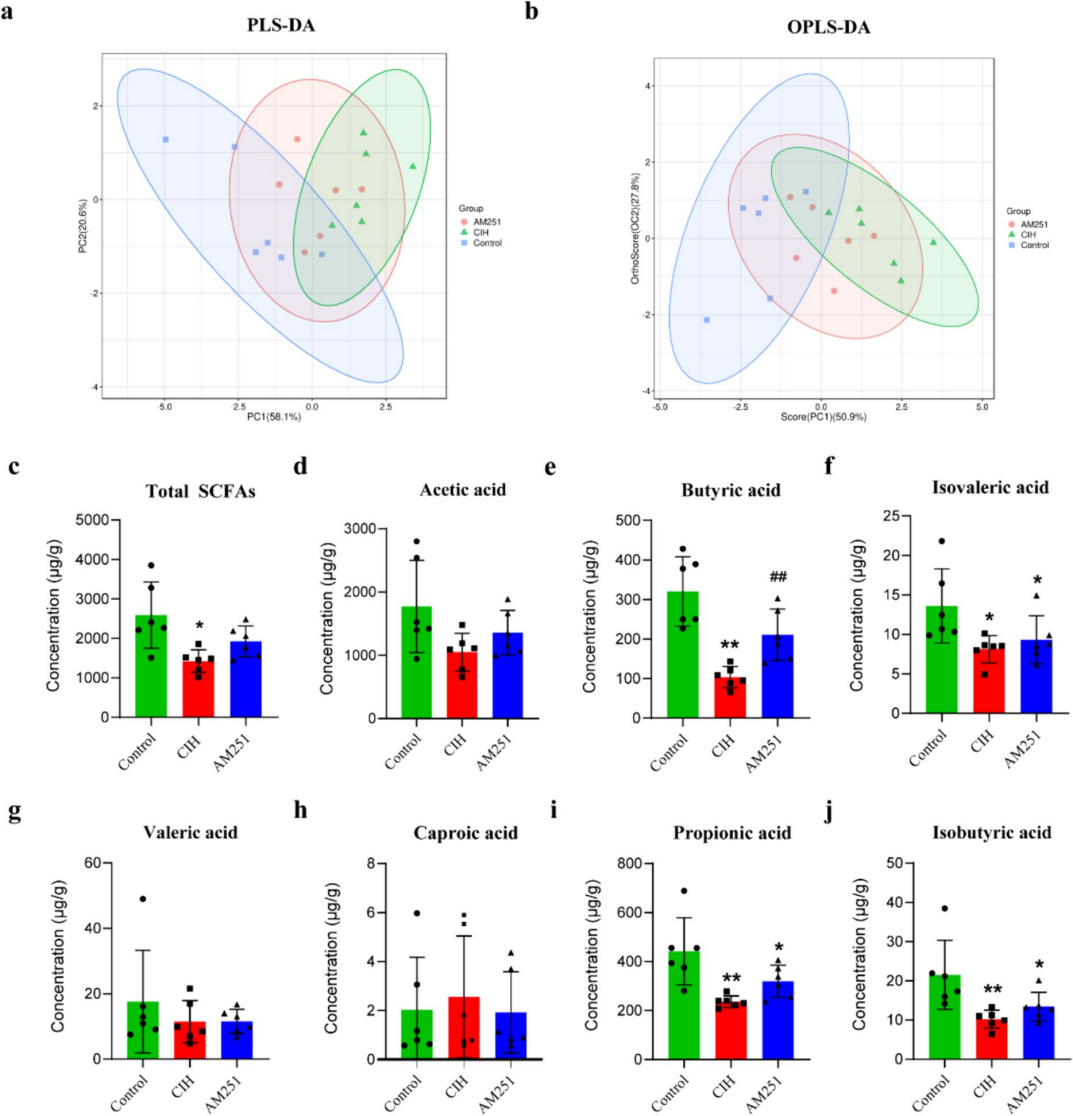
CB1 receptor inhibition may ameliorate SCFA metabolic disorders induced by CIH. **a** Partial least squares discrimination analysis (PLS-DA). **b** Score plot of the OPLS-DA model. **c**–**j** Concentrations of SCFAs in each group. **c** Total SCFAs. **d** Acetic acid. **e** Butyric acid. **f** Isovaleric acid. **g** Valeric acid. **h** Caproic acid. **i** Propionic acid. **j** Isobutyric acid. *P < 0.05, **P < 0.01, compared to the Control group; ^#^*P* < 0.05, ^##^*P* < 0.01, compared to the CIH group

To probe the effect of inhibiting CB1 receptor on fecal SCFA content in CIH-exposed mice, we used multivariate statistical analysis to investigate the degree of aggregation and dispersion between samples (Fig. 7a, b). SCFA analysis showed that the CIH group had substantially decreased levels of total SCFAs contents, butyric acid, propionic acid, isovaleric acid, and isobutyric acid than the Control group. Acetic and valeric acid contents were also decreased (Fig. 7c–j). Conversely, upon treatment with AM251, butyric acid content was significantly increased (Fig. 7e).

### Inhibition of CB1 receptor promotes the expression of tight junction proteins and reduces endotoxemia

**Fig. 8:**
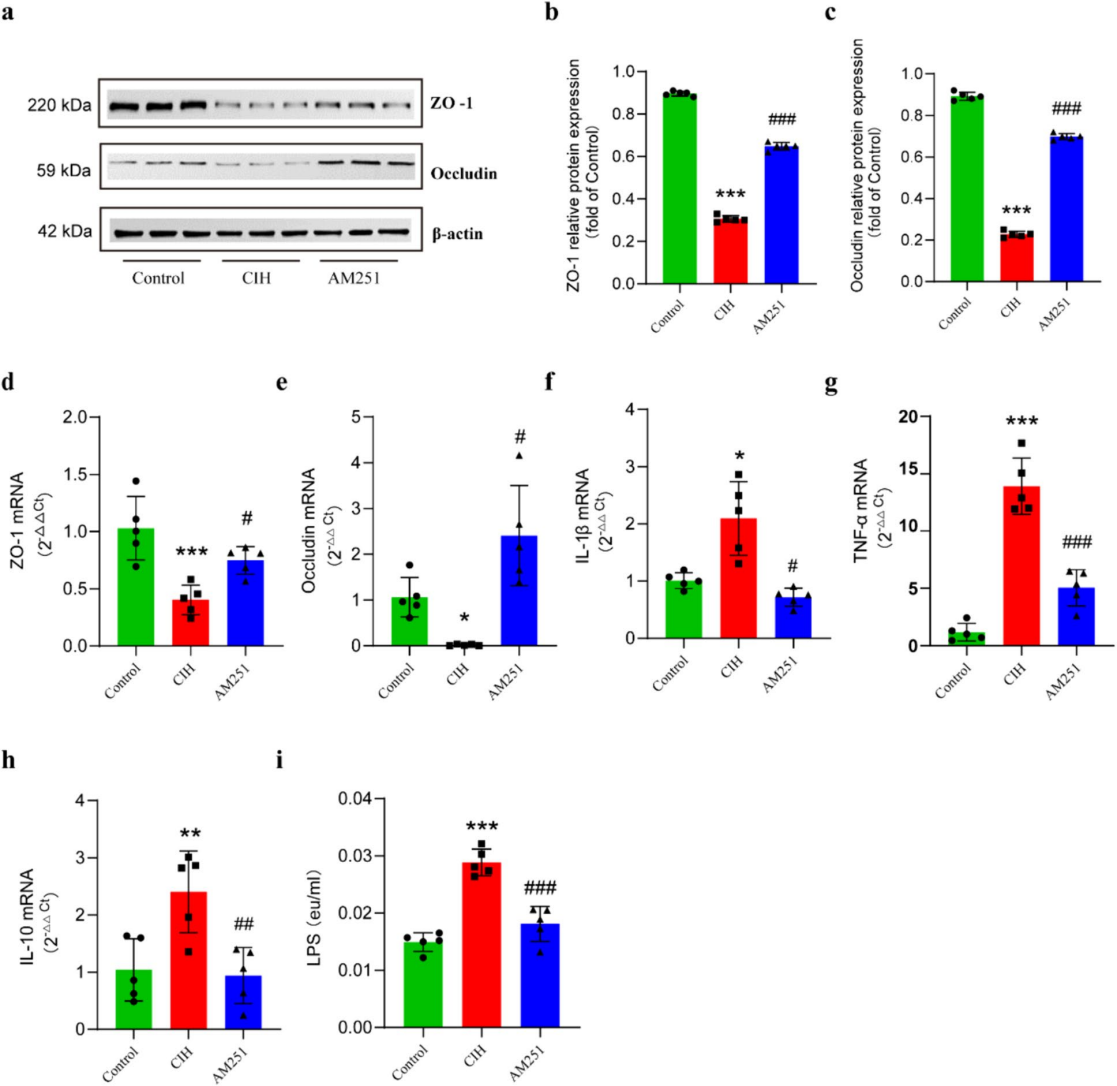
Inhibition of CB1 receptor promotes the production of intestinal tight junction proteins under CIH conditions and reduces intestinal inflammation and metabolic endotoxemia. **a**–**c** Western blot to detect the expression of ZO-1 and Occludin in colon. **d**, **e** RT-PCR to detect the mRNA expression of ZO-1 and Occludin in colon. **f**–**h** RT-PCR to detect IL-1β (**f**), TNF-α (**g**), and IL-10 mRNA (**h**). **i** Serum LPS level detected by ELISA (eu, ELISA units). *P < 0.05, **P < 0.01, compared to the Control group; ^#^*P* < 0.05, ^##^*P* < 0.01, compared to the CIH group

To investigate if CB1 receptor inhibition contributed to the compromised intestinal barrier function and inflammatory response in CIH-exposed mice, we examined the protein and gene expressions of tight junction protein and inflammatory cytokines in colon tissues by western blot and RT-PCR. We observed that the levels of expression of ZO-1 and Occludin protein and mRNA in mice in the CIH group were both considerably reduced than those in the Control group, whereas the levels of ZO-1 and Occludin treated with AM251 were significantly increased (Fig. 8a–e). In addition, the mRNA levels of the proinflammatory factors IL-1β and TNF-α were significantly decreased, and the anti-inflammatory factor IL-10 was significantly increased in the AM251 group as opposed to the CIH group (Fig. 8f–h). These findings suggest that CB1 receptors are involved in intestinal barrier degradation and colon inflammation caused by CIH. Taken together, these data suggest that CIH exposure causes colon barrier dysfunction and aggravates colon inflammatory responses in mice and that inhibiting CB1 receptors is critical for maintaining intestinal barrier integrity and suppressing colon inflammation.

Metabolic endotoxemia is usually caused by the imbalance of intestinal flora, and endotoxins also significantly participate in the multi-organ damage caused by OSA. Therefore, to evaluate the effect of inhibiting the CB1 receptor on metabolic endotoxemia in CIH-exposed mice, serum LPS levels were assessed. The data demonstrated that the serum LPS level of the mice in the CIH group was substantially higher than that of the Control group, and AM251 intervention could significantly reduce the LPS level of CIH mice (Fig. 8i).

### Relationship between intestinal flora and physiological indexes

**Fig. 9:**
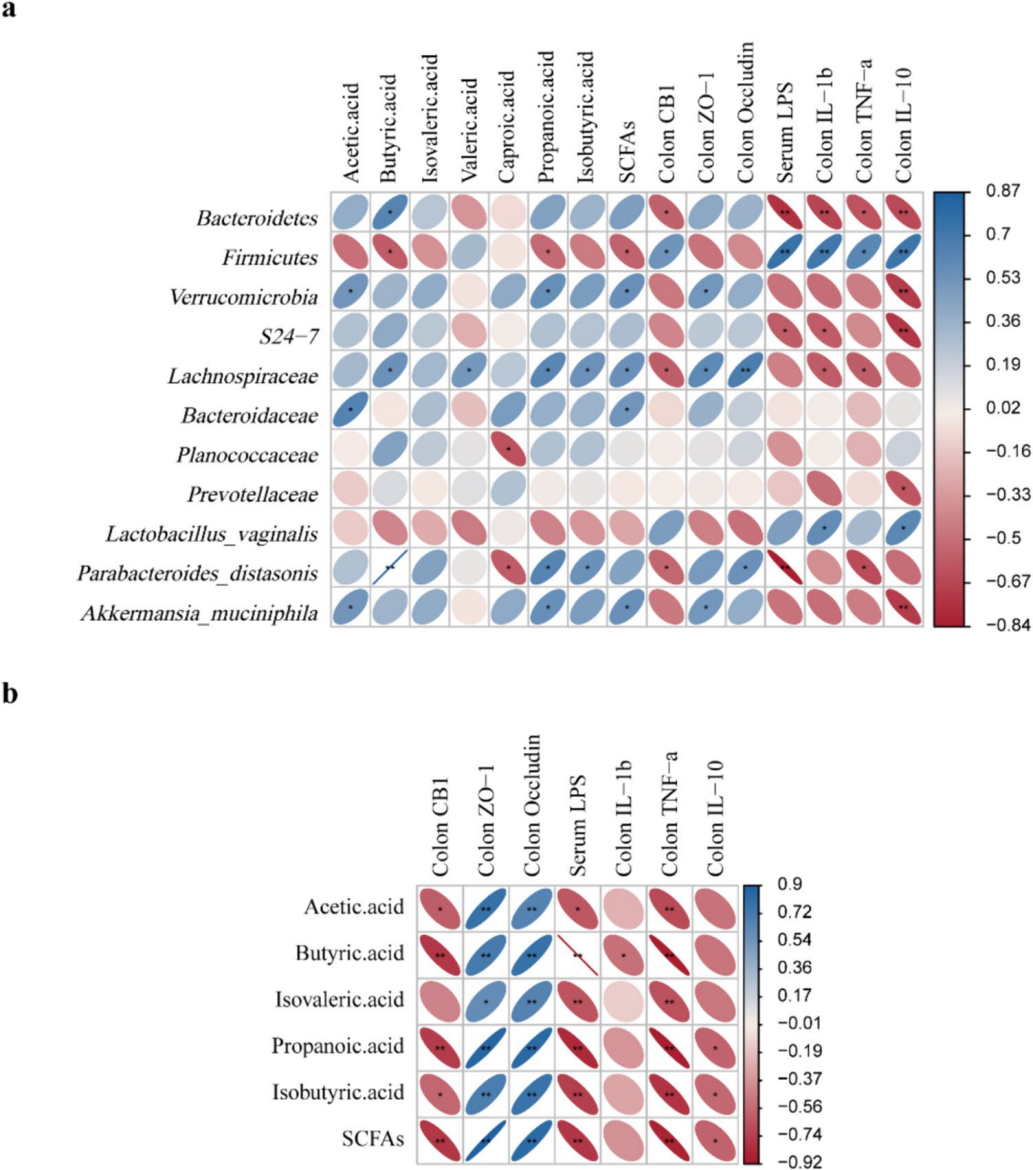
The relationship between intestinal flora and physiological indexes. **a** Heat map analysis showed the correlation between intestinal flora and physiological indexes. **b** The relationship between SCFA and cytokines. The depth of the color indicates the strength of the correlation: red indicates a positive correlation, blue indicates a negative correlation, and white indicates no correlation. *P < 0.05, **P < 0.01

To examine the relationship between physiological indexes and intestinal flora, spearman correlation analysis was carried out based on experimental parameters. The findings revealed that *Bacteroidetes* and *Lachnospiraceae* were positively correlated with butyric acid, whereas *Firmicutes* was negatively correlated with it. Additionally, *Bacteroidetes*, *Lachnospiraceae*, and *Parabacteroides_distasonis* were found to be negatively correlated with colon CB1 protein. The findings indicated that with regard to intestinal tight junction proteins, *Verrucomicrobia*, *Lachnospiraceae*, and *Akkermansia_muciniphila* were positively correlated with ZO-1 and *Lachnospiraceae* and *Parabacteroides_distasonis* were positively correlated with Occludin. Similarly, *Bacteroidetes*, *S24-7*, and *Parabacteroides_distasonis* were negatively correlated with serum LPS but positively correlated with *Firmicutes* (Fig. 9a).

Next, we examined the connection between SCFA and cytokines in more detail. It was discovered that acetic acid, butyric acid, propionic acid, isobutyric acid, and SCFAs were highly negatively correlated to CB1, but had a significant positive correlation with ZO-1 and Occludin. Serum LPS and TNF-α levels were negatively correlated with acetic acid, isovaleric acid, propionic acid, isobutyric acid, and SCFAs (Fig. 9b).

## Discussion

As far as we know, this is the first study to clarify how CIH, one of the prominent pathophysiological characteristics of OSA, induces destruction of intestinal barrier integrity through abnormal expression of CB1 receptor. In this study, utilizing the CIH model, we discovered that the overexpression of the colonic CB1 receptor caused by CIH is a pivotal mechanism behind the disruption of the colonic intestinal barrier induced by CIH. Additionally, administration of CB1 receptor antagonist reversed the impairment of the colonic intestinal barrier caused by CIH.

In this study, our results showed that CIH mice exhibited damaged intestinal mucosal barrier, including disrupted tight junction, severely swollen mitochondria, shed microvilli, and infiltrated inflammatory cells, as evidenced by decreased expression of ZO-1 and Occludin. Additionally, CIH led to the overexpression of CB1 receptor in colon tissue. Meanwhile, AM251 improved the intestinal barrier damage caused by CIH. AM251 is a powerful selective antagonist of CB1 receptor with greater binding affinity and selectivity (Lan et al. 1999). By treating CIH-exposed mice with CB1 receptor antagonist AM251, we found that tight junctions between intestinal mucosa cells were restored; intracellular mitochondrial swelling, inflammatory cell infiltrating, and microvilli shedding were relieved; and ZO-1 and Occludin expression levels also increased significantly. In this study, CB1 receptor inhibition alleviated CIH-induced colon injury by elevating tight junction protein expression levels and reducing mitochondrial swelling and inflammatory cell infiltration in colon.

The dysregulated intestinal mucosal barrier function can allow numerous toxins to get through the bloodstream and lead to chronic inflammation due to the impaired intestinal barrier. Therefore, the degree of intestinal mucosal injury is closely related to inflammation (Williams 2001). TNF-α is a factor secreted by activated macrophages to initiate an inflammatory response, which can promote the proliferation and differentiation of macrophages, coordinate the body’s inflammatory response, elevate the expression of pro-inflammatory factors IL-1β and IL-6, and cause various inflammatory cells to infiltrate intestinal tissues to promote the development of intestinal inflammation and aggravate intestinal barrier damage. Inflammation has been shown to be closely associated with most OSA-related conditions, such as hypertension, coronary heart disease, obesity, and diabetes (Turnbull et al. 2020; Arnaud et al. 2020; Sanderson et al. 2020; Campos-Rodriguez et al. 2021; Wali et al. 2021). Inhibiting CB1 receptor has been reported to reduce liver and adipose tissue inflammation in obese mice (Wang et al. 2011). The application of AM251 in diabetic mice may lower the expression levels of inflammatory factors TNF-α and IL-6 in the liver (Chen et al. 2020). In line with earlier discoveries, our findings also reveal that inhibiting the CB1 receptor reduces the IL-1β and TNF-α expression levels and increases level of the anti-inflammatory factor IL-10 in CIH-exposed mice. Thus, CB1 receptor inhibition exerts anti-inflammatory effects in CIH-exposed mice by modulating inflammatory mediators.

Furthermore, in the process of exploring the colon inflammation caused by CIH, our study still found that CIH leads to the translocation of LPS from the intestine to the blood, and further elucidated that CB1 receptor may be involved in this process. LPS is an endotoxin part of gram-negative bacteria’s cell wall and is the primary means by which it can cause harm. It plays an integral part in inducing related metabolic diseases, as such it is named “metabolic endotoxemia” (Cani et al. 2007). LPS produced by intestinal bacteria leak from the intestine to the systemic circulatory system, where they trigger the release of other inflammatory mediators, further leading to systemic inflammation (Tucureanu et al. 2018). Our study found that CIH exposure increased serum LPS levels in mice, and CB1 receptor inhibition significantly reduced LPS level. Therefore, we believe that inhibiting the CB1 receptor mitigated metabolic endotoxin in CIH-exposed mice.

It is well known that intestinal flora is one of the major components of the intestinal barrier. The disturbance of flora will cause a series of adverse consequences to the intestinal tract, such as increased toxic substances in the intestinal cavity, decreased SCFAs, damaged intestinal mucosal epithelial cells as well as degraded mucin, and abnormal immune cell activation, which may ultimately result in the destruction of intestinal barrier function. By constructing a CIH model, this study found that the intestinal flora disorder caused by CIH includes a decrease in alpha diversity, an increase in the ratio of F to B, and a decrease in the abundance of bacteria that produce SCFAs. The abovementioned changes in the flora were observed in patients with OSA (Ko et al. 2019; Wang et al. 2022a). The bacteria in the colon are mainly composed of *Bacteroides* and *Firmicutes* (Dominguez-Bello et al. 2011). The increase in the abundance of *Firmicutes* and the decrease in the abundance of *Bacteroidetes* will lead to an increase in the F/B ratio. Previous studies have shown that the ratio of F to B is a marker of intestinal dysbiosis and is closely related to intestinal immune inflammation (Min et al. 2019; Li et al. 2021; Tilahun et al. 2022). *Bacteroidetes* are considered to be the source of the next generation of probiotics and can improve various intestinal diseases (Ma et al. 2020). Interestingly, this study can change the intestinal dysbiosis caused by CIH to some extent by using the CB1 receptor antagonist AM251. Inhibiting CB1 receptor can significantly reduce the quantity of *Firmicutes* and the F to B ratio while increasing the quantity of *Bacteroidetes*. More importantly, we found that *Firmicutes* and colon CB1, serum LPS, and inflammatory factors had a positive correlation, while it was negatively correlated to SCFA such as butyric acid and propanoic acid. The phylum *Bacteroidetes* is negatively correlated with colon CB1, serum LPS, and inflammatory factors. At the family level, we found that CB1 receptor inhibition elevated the quantity of SCFA-producing bacteria *Lachnospiraceae*, *Ruminococcaceae*, and *Lachnospiraceae* and had a negative correlation to CB1. At the species level, inhibition of the CB1 receptor decreased the number of *Lactobacillus_vaginalis* and *Akkermansia_muciniphil* and increased *Parabacteroides_distasonis* in CIH-exposed mice. Therefore, we hypothesized that CB1 receptor inhibition provides deep protection of the gut microbiota in CIH-exposed mice, preventing bacterial community imbalance.

An essential metabolite of intestinal flora, SCFA modulates appetite regulation, glucose and lipid metabolism regulation, intestinal barrier integrity, oxidative stress, inflammation, and immune system homeostasis (Jiao et al. 2021; Liu et al. 2021). The production of bacterial butyrate is critical for regulating intestinal microecological stability, as this class of butyric acid is considered to be the primary source of energy in intestinal epithelial cells (Sonnenburg et al. 2010). Previous data have shown that butyric acid enhances the function of the intestinal barrier by upregulating tight junction protein expression and reduces LPS release into the bloodstream, and intestinal inflammatory response (Peng et al. 2009; Moreno-Indias et al. 2014). Thus, the synthesis of bacterial SCFA is crucial for the regulation of intestinal microecological stability. Recent studies have found that the components of acetic acid, butyric acid, and propionic acid in serum were substantially reduced in patients presenting severe OSA (Zhang et al. 2022). Our research revealed that the components of butyric acid, isovaleric acid, propionic acid, and isobutyric acid were considerably decreased in CIH-exposed mice, while CB1 receptor inhibition can significantly increase butyric acid concentration. Furthermore, correlation analysis also revealed a positive correlation between the butyric acid level and *Lachnospiraceae*, ZO-1, and Occludin, but negatively correlated with colon CB1, IL-1β, and TNF-α.

Although this study found that CB1 receptor antagonist can improve the colonic injury caused by CIH and further explored the potential mechanisms, there are still some limitations in this study. Firstly, this study lacks direct evidence of the role of CB1 receptor in improving intestinal flora imbalance and colonic pathological injury. Secondly, the relationship between CB1 receptor and colon injury was not further validated in cell experiments. Finally, CIH can only simulate the pathological changes caused by CIH in patients with OSA, and cannot fully simulate the pathophysiological processes in patients with OSA. Meanwhile, the results of animal experiments cannot be extrapolated to patients with OSA. Therefore, the application of CB1 receptor antagonist in the treatment of patients with OSA and colonic injury needs further confirmation in multi-center clinical trials.

In conclusion, CBI receptors play a crucial role in colon injury caused by CIH. CIH leads to increased expression of CB1 receptor in colon tissues, damage to the colonic mucosa, decreased expression of tight junction proteins, disruption of gut microbiota, increased levels of inflammatory mediators in the gut, and LPS translocation. Moreover, inhibiting CB1 receptor is an effective way to alleviate colon injury caused by CIH.

## Data Availability

Upon reasonable request, the corresponding author will provide the datasets created during the current work. The raw sequence data of microbiota that corroborate our study’s conclusions have been added to the NCBI SRA with accession number PRJNA1052947.
